# The genome sequence of petty spurge, *Euphorbia peplus* L. (Euphorbiaceae)

**DOI:** 10.12688/wellcomeopenres.24030.1

**Published:** 2025-04-25

**Authors:** Maarten J. M. Christenhusz, Michael F. Fay, Ilia J. Leitch, Royal Botanic Gardens Kew Genome Acquisition Lab, Darwin Tree of Life Barcoding collective, Plant Genome Sizing collective, Wellcome Sanger Institute Tree of Life Management, Samples and Laboratory team, Wellcome Sanger Institute Scientific Operations: Sequencing Operations, Wellcome Sanger Institute Tree of Life Core Informatics team, Tree of Life Core Informatics collective, Darwin Tree of Life Consortium

**Affiliations:** 1 Royal Botanic Gardens Kew, Richmond, England, UK; 2 Curtin University, Perth, Western Australia, Australia

**Keywords:** Euphorbia peplus, petty spurge, genome sequence, chromosomal, Malpighiales

## Abstract

We present a genome assembly from a specimen of *Euphorbia peplus* (petty spurge; Streptophyta; Magnoliopsida; Malpighiales; Euphorbiaceae). The genome sequence has a total length of 277.10 megabases. Most of the assembly is scaffolded into 8 chromosomal pseudomolecules. We also assembled six multipartite mitochondrial molecules and one plastid genome.

## Species taxonomy

Eukaryota; Viridiplantae; Streptophyta; Streptophytina; Embryophyta; Tracheophyta; Euphyllophyta; Spermatophyta; Magnoliopsida; Mesangiospermae; eudicotyledons; Gunneridae; Pentapetalae; rosids; fabids; Malpighiales; Euphorbiaceae; Euphorbioideae; Euphorbieae; *Euphorbia*; *Euphorbia* subgen. *Esula*; *Euphorbia* sect. *Tithymalus*; *Euphorbia peplus* L. (NCBI:txid38846)

## Background

**Figure 1.**
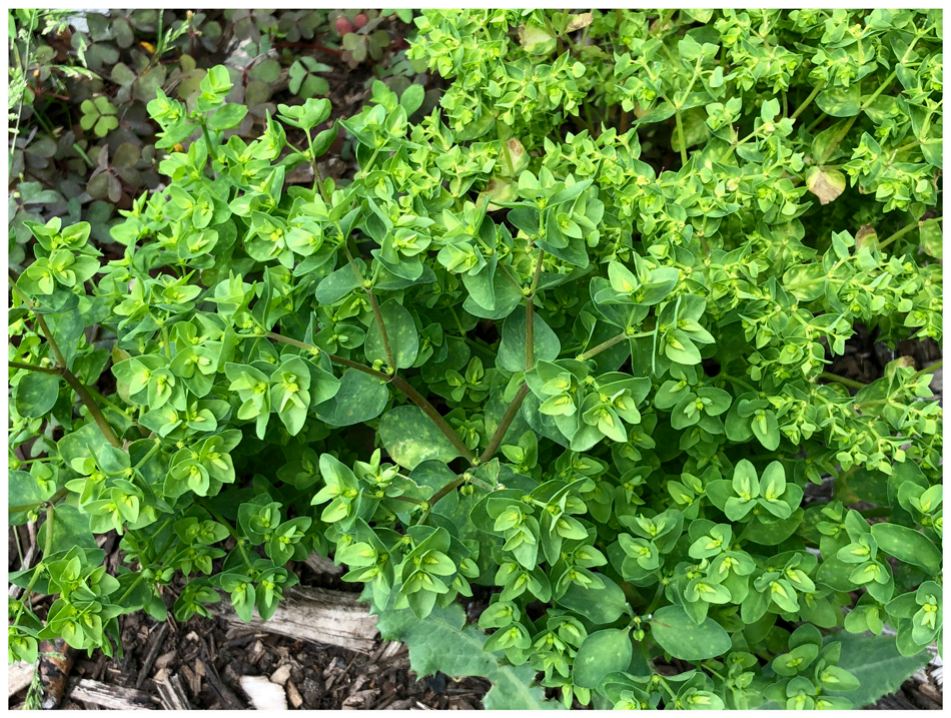
Photograph of the *Euphorbia peplus* (ddEupPepu3) from which samples were taken for genome sequencing.

Petty spurge ( *Euphorbia peplus*, Euphorbiaceae) is an annual, weedy plant, commonly found in arable land, gardens, along paths, pavements and waste places. It prefers well-drained, nutrient-rich soils on sunny, warm locations. While it can grow up to 30 cm tall, it is usually much smaller, especially in exposed sites. The entire plant is pale green with lanceolate to ovate, pointed leaves and three-rayed inflorescences ( Figure 1). Like other spurges, the flowers are minute and reduced, but several male flowers and a single female flower are surrounded by fused bracts called a cyathium, which are topped by glands. The glands of this species are kidney-shaped with two thin horns.

In the UK, this species is an archaeophyte (e.g. Stace, 2010), introduced from southern Europe in ancient times as a medicinal plant. It is now widely naturalised outside its native range and is one of the most common garden weeds across the temperate regions of the world, even reaching remote Easter Island ( POWO, 2024; Zizka, 1991).

When damaged, the plant emits a milky latex, which is toxic to rapidly replicating cells. In humans this has traditionally been used for treating common skin lesions, including those caused by exposure to UV light from the sun, and basal cell carcinomas. The active ingredient, the diterpene ester ingenol mebutate, which is cytotoxic and is currently used medicinally for the treatment of squamous cell skin cancers and actinic keratoses ( Braun *et al.*, 2014; Siller *et al.*, 2009). In preclinical trials, the data suggest that ingenol mebutate may also be effective in treating other types of cancers such as pancreatic, colorectal and epithelial cancers (reviewed in Shafombabi *et al.* 2025). However, there are safety concerns due to its cytotoxicity, so ongoing research is exploring how to minimize these risks, including the exploration of advanced delivery methods to enable its potential as an anticancer treatment to be expanded.

Despite a wide diversity of chromosome numbers reported for the genus *Euphorbia* (2 *n* = 12 to ~200; Hans, 1973; Perry, 1943) arising from polyploidy, aneuploidy and structural rearrangements, *E. peplus* has so far only been reported to be diploid with 2 *n* = 2 *x* = 16, based on a single chromosome count reported from the UK ( Al-Bermani *et al.*, 1993; Perry, 1943) and several counts from mainland Europe (e.g. see Hans, 1973).

For the Darwin Tree of Life project, plants were collected from flower beds at the Royal Botanic Gardens, Kew. During the COVID-19 pandemic lockdowns, the beds had not been weeded, and petty spurge grew there in abundance. Here we present a high-quality genome of *E. peplus*. It complements the previously published chromosome level genome assembly for the same species, but sourced from a wild-grown individual plant in the Cornell Botanical Gardens, USA ( Johnson *et al.*, 2023). Both resources, together with a chromosome level genome assembly of a related species, *E. lathyris* ( Wang *et al.*, 2021), will be useful for enabling further studies into its medicinal properties and latex production.

## Genome sequence report

### Sequencing data

The genome of a specimen of *Euphorbia peplus* was sequenced using Pacific Biosciences single-molecule HiFi long reads, generating 22.40 Gb (gigabases) from 1.57 million reads. GenomeScope analysis of the PacBio HiFi data estimated the haploid genome size at 355.40 Mb, with a heterozygosity of 0.06% and repeat content of 46.13%. These values provide an initial assessment of genome complexity and the challenges anticipated during assembly. Based on this estimated genome size, the sequencing data provided approximately 60.0x coverage of the genome. Using flow cytometry, the genome size (1C-value) was estimated to be 0.4 pg, equivalent to 390 Mb. Hi-C sequencing produced 98.03 Gb from 649.22 million reads. Table 1 summarises the specimen and sequencing information.

**Table 1.** Specimen and sequencing data for *Euphorbia peplus*.

Project information			
**Study title**	Euphorbia peplus		
**Umbrella BioProject**	PRJEB52211		
**Species**	*Euphorbia peplus*		
**BioSample**	SAMEA7521821		
**NCBI taxonomy ID**	38846		
Specimen information			
**Technology**	**ToLID**	**BioSample accession**	**Organism part**
**PacBio long read sequencing**	ddEupPepu3	SAMEA7521875	leaf
**Hi-C sequencing**	ddEupPepu3	SAMEA7521873	leaf
**RNA sequencing**	ddEupPepu3	SAMEA7521875	leaf
Sequencing information			
**Platform**	**Run accession**	**Read count**	**Base count (Gb)**
**Hi-C Illumina NovaSeq 6000**	ERR9580485	6.49e+08	98.03
**PacBio Sequel IIe**	ERR9630945	1.57e+06	22.4
**RNA Illumina HiSeq 4000**	ERR9580484	4.47e+07	6.74

### Assembly statistics

The primary haplotype was assembled, and contigs corresponding to an alternate haplotype were also deposited in INSDC databases. The assembly was improved by manual curation, which corrected 5 misjoins or missing joins. The interventions increased the scaffold count by 90.91% and decreased the scaffold N50 by 46.68%. The final assembly has a total length of 277.12 Mb in 14 scaffolds, with 5 gaps, and a scaffold N50 of 33.77 Mb ( Table 2).

**Table 2.** Genome assembly data for *Euphorbia peplus*, ddEupPepu3.1

Genome assembly		
Assembly name	ddEupPepu3.1	
Assembly accession	GCA_964200815.1	
*Alternate haplotype accession*	*GCA_964200835.1*	
Assembly level for primary assembly	chromosome	
Span (Mb)	277.12	
Number of contigs	19	
Number of scaffolds	14	
Longest scaffold (Mb)	41.2	
Assembly metric	Measure	*Benchmark*
Contig N50 length	27.11 Mb	*≥ 1 Mb*
Scaffold N50 length	33.77 Mb	*= chromosome N50*
Consensus quality (QV)	Primary: 54.2; alternate: 47.4; combined: 53.9	*≥ 40*
*k*-mer completeness	Primary: 97.97%; alternate: 1.18%; combined: 98.49%	*≥ 95%*
BUSCO *	C:97.6%[S:94.4%,D:3.2%], F:0.5%,M:1.9%,n:2,326	*S > 90%; D < 5%*
Percentage of assembly mapped to chromosomes	99.54%	*≥ 90%*
Organelles	Mitochondrial genome: six separate molecules; Plastid genome: 159.6 kb	*complete single alleles*

* Assembly metric benchmarks are adapted from Rhie *et al.* (2021) and the Earth BioGenome Project Report on Assembly Standards September 2024. ** BUSCO scores based on the eudicotyledons_odb10 BUSCO set using version 5.4.3. C = complete [S = single copy, D = duplicated], F = fragmented, M = missing, n = number of orthologues in comparison. A full set of BUSCO scores is available at https://blobtoolkit.genomehubs.org/view/Euphorbia_peplus/dataset/GCA_964200815.1/busco.

**Figure 2.**
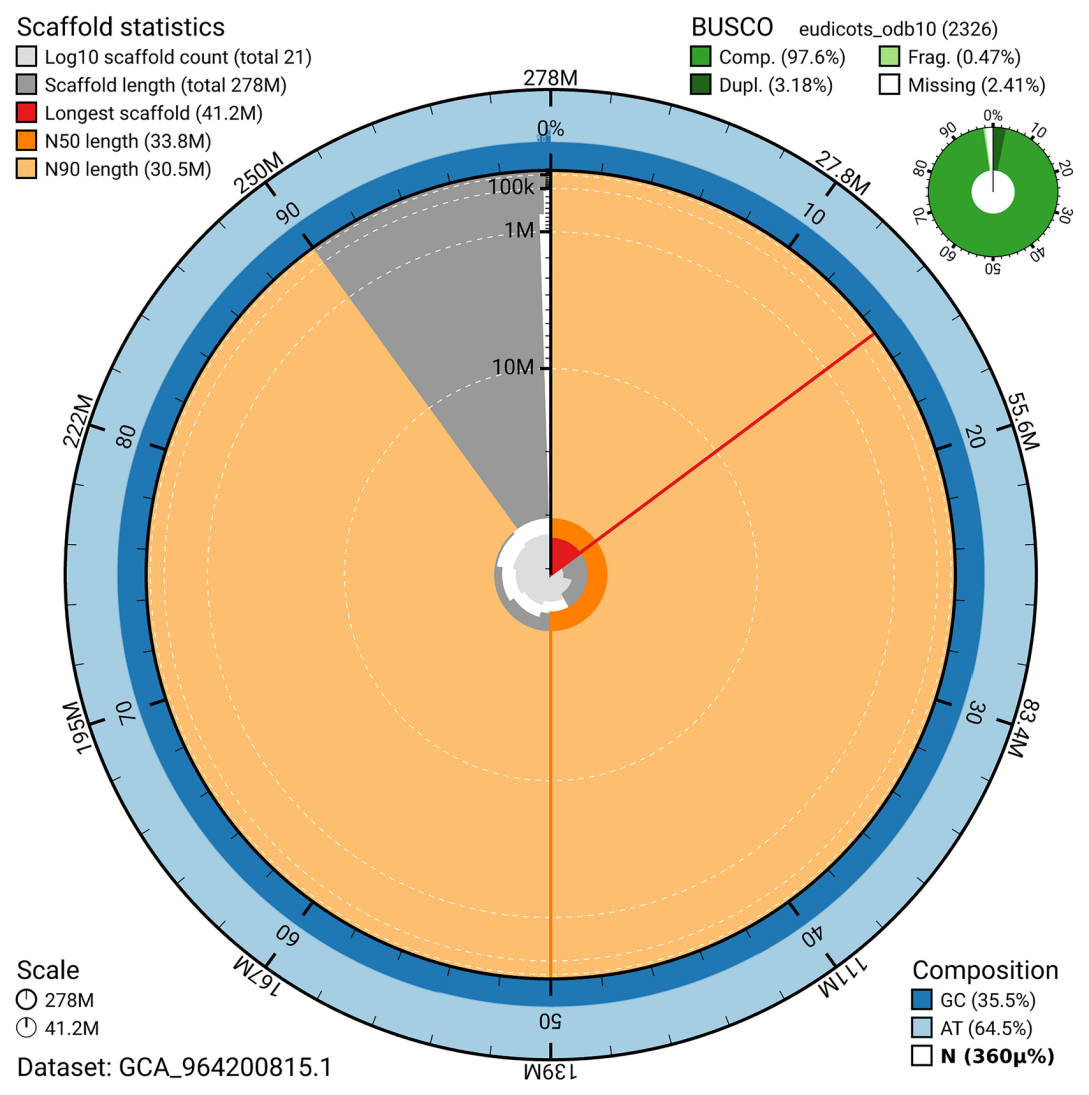
Snail plot summary of assembly statistics for assembly ddEupPepu3.1: metrics. The BlobToolKit snail plot provides an overview of assembly metrics and BUSCO gene completeness. The circumference represents the length of the whole genome sequence, and the main plot is divided into 1,000 bins around the circumference. The outermost blue tracks display the distribution of GC, AT, and N percentages across the bins. Scaffolds are arranged clockwise from longest to shortest and are depicted in dark grey. The longest scaffold is indicated by the red arc, and the deeper orange and pale orange arcs represent the N50 and N90 lengths. A light grey spiral at the centre shows the cumulative scaffold count on a logarithmic scale. A summary of complete, fragmented, duplicated, and missing BUSCO genes in the eudicotyledons_odb10 set is presented at the top right. An interactive version of this figure is available at https://blobtoolkit.genomehubs.org/view/GCA_964200815.1/dataset/GCA_964200815.1/snail.

**Figure 3.**
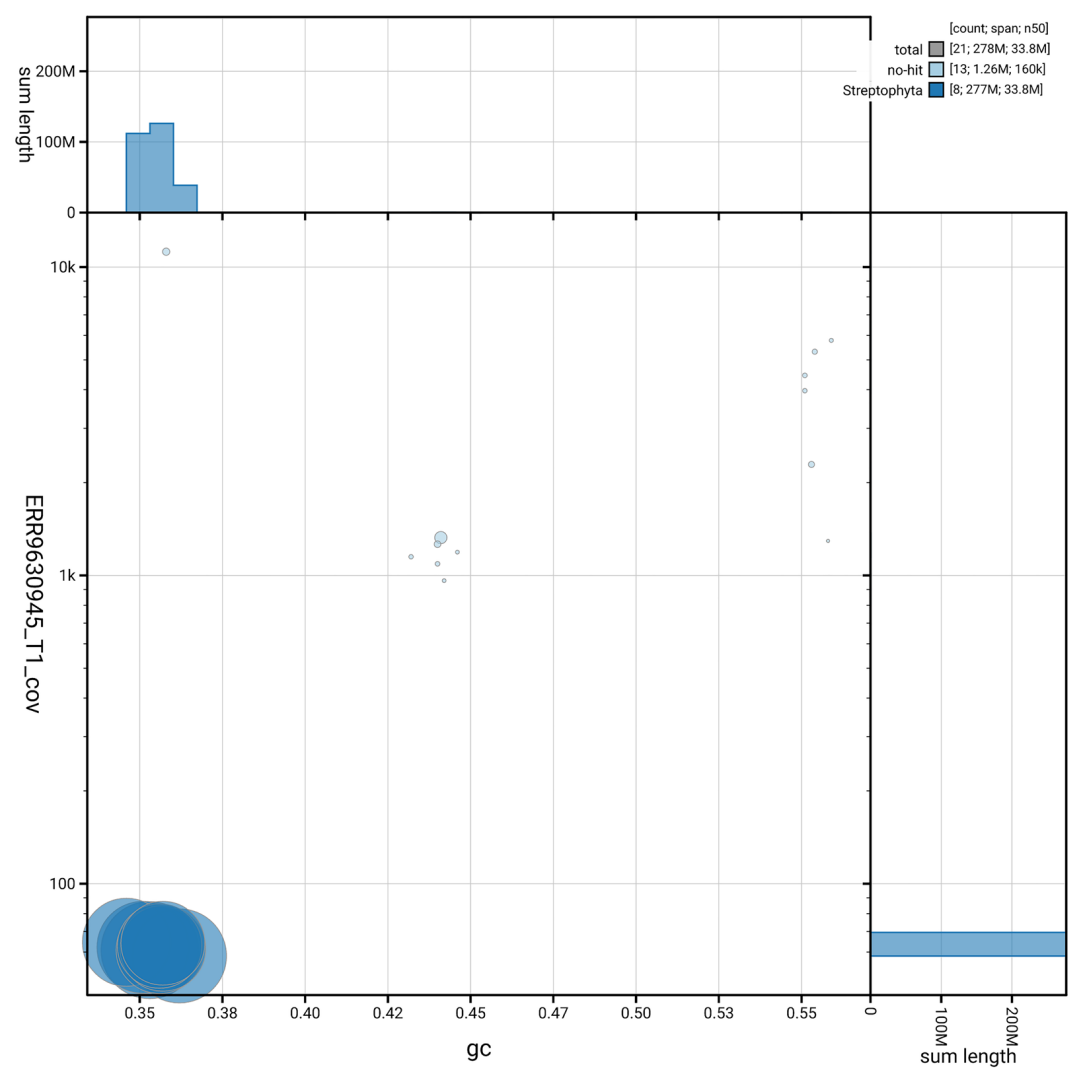
BlobToolKit blob plot for assembly ddEupPepu3.1: BlobToolKit GC-coverage plot showing sequence coverage (vertical axis) and GC content (horizontal axis). The circles represent scaffolds, with the size proportional to scaffold length and the colour representing phylum membership. The histograms along the axes display the total length of sequences distributed across different levels of coverage and GC content. An interactive version of this figure is available at https://blobtoolkit.genomehubs.org/view/GCA_964200815.1/dataset/GCA_964200815.1/blob.

**Figure 4.**
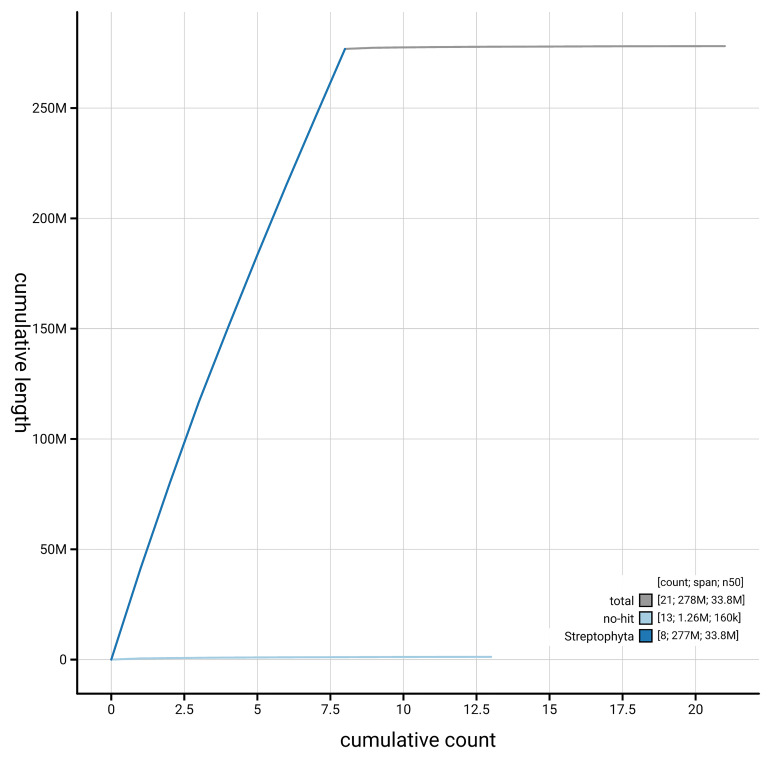
BlobToolKit cumulative sequence plot. The grey line shows cumulative length for all scaffolds. Coloured lines show cumulative lengths of scaffolds assigned to each phylum using the buscogenes taxrule. An interactive version of this figure is available at https://blobtoolkit.genomehubs.org/view/GCA_964200815.1/dataset/GCA_964200815.1/cumulative.

The snail plot in Figure 2 provides a summary of the assembly statistics, indicating the distribution of scaffold lengths and other assembly metrics. Figure 3 shows the distribution of scaffolds by GC proportion and coverage. Figure 4 presents a cumulative assembly plot, with separate curves representing different scaffold subsets assigned to various phyla, illustrating the completeness of the assembly.

**Figure 5.**
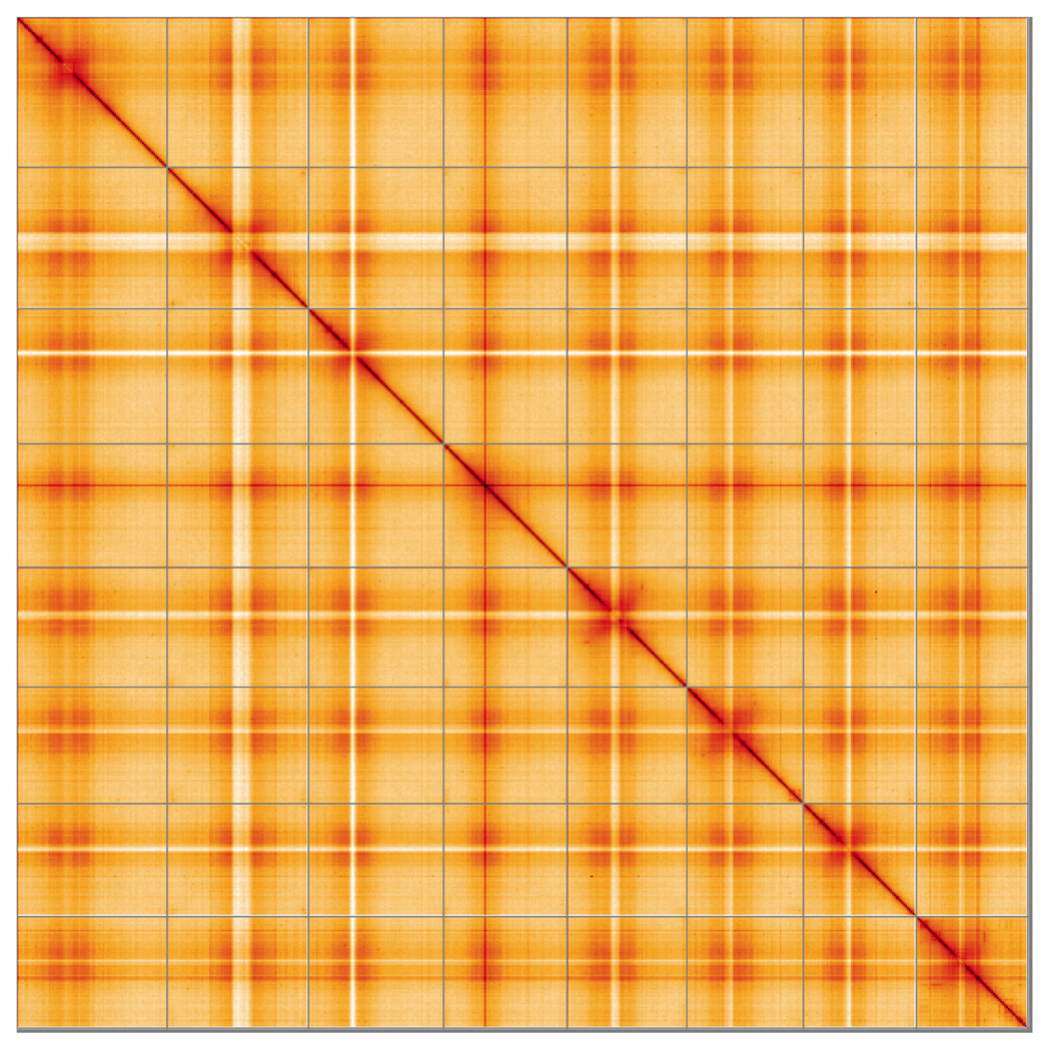
Genome assembly of *Euphorbia peplus*, ddEupPepu3.1: Hi-C contact map of the ddEupPepu3.1 assembly, visualised using HiGlass. Chromosomes are shown in order of size from left to right and top to bottom. Darker shades indicate more frequent physical contacts between regions, while lighter areas represent fewer contacts. An interactive version of this figure may be viewed at https://genome-note-higlass.tol.sanger.ac.uk/l/?d=NjzabIgvS9aw_nejOLH5XQ.

Most of the assembly sequence (99.54%) was assigned to 8 chromosomal-level scaffolds. These chromosome-level scaffolds, confirmed by Hi-C data, are named according to size ( Figure 5; Table 3).

**Table 3.** Chromosomal pseudomolecules in the genome assembly of *Euphorbia peplus*, ddEupPepu3.

INSDC accession	Name	Length (Mb)	GC%
OZ123146.1	1	41.2	35.5
OZ123147.1	2	38.69	36.0
OZ123148.1	3	36.98	35.0
OZ123149.1	4	33.77	34.5
OZ123150.1	5	32.81	35.5
OZ123151.1	6	31.94	35.5
OZ123152.1	7	30.89	35.5
OZ123153.1	8	30.53	35.5
OZ123160.1	Pltd	0.16	36.0
OZ123154.1	MT1	0.52	44.0
OZ123155.1	MT2	0.03	44.5
OZ123156.1	MT3	0.03	44.5
OZ123157.1	MT4	0.13	44.0
OZ123158.1	MT5	0.04	43.0
OZ123159.1	MT6	0.05	44.0

The mitochondrial and plastid genomes were also assembled. These sequences are included as contigs in the multifasta file of the genome submission and as standalone records in GenBank.

### Assembly quality metrics

The estimated Quality Value (QV) and *k*-mer completeness metrics, along with BUSCO completeness scores, were calculated for each haplotype and the combined assembly. The QV reflects the base-level accuracy of the assembly, while *k*-mer completeness indicates the proportion of expected *k*-mers identified in the assembly. BUSCO scores provide a measure of completeness based on benchmarking universal single-copy orthologues.

The primary haplotype has a QV of 54.2, and the combined primary and alternate assemblies achieve an estimated QV of 53.9. The *k*-mer recovery for the primary haplotype is 97.97%, and for the alternate haplotype 1.18%; the combined primary and alternate assemblies have a *k*-mer recovery of 98.49%. BUSCO analysis using the eudicots_odb10 reference set ( *n* = 2,326) identified 97.6% of the expected gene set (single = 94.4%, duplicated = 3.2%).

Table 2 provides assembly metric benchmarks adapted from Rhie *et al.* (2021) and the Earth BioGenome Project (EBP) Report on Assembly Standards September 2024. The primary assembly achieves the EBP reference standard of **7.C.Q54.**

## Methods

### Sample acquisition, DNA barcoding and genome size estimation

A sample of *Euphorbia peplus* (specimen ID KDTOL10012, ToLID ddEupPepu3) was collected from the Royal Botanic Gardens, Kew, Surrey, UK (latitude 51.47, longitude –0.30) on 2020-08-05. The specimen was collected and identified by Maarten J. M. Christenhusz (collection number MC9009) and preserved by freezing at –80 °C. The herbarium voucher associated with the sequenced plant (K001400680) is deposited in the herbarium of RBG Kew (K).

The initial species identification was verified by an additional DNA barcoding process following the framework developed by Twyford *et al*. (2024). Part of the plant specimen was preserved in silica gel desiccant ( Chase & Hills, 1991). DNA was extracted from the dried specimen, then PCR was used to amplify standard barcode regions. The resulting amplicons were sequenced and compared to public sequence databases including GenBank and the Barcode of Life Database (BOLD). The barcode sequences for this specimen are available on BOLD ( Ratnasingham & Hebert, 2007). Following whole genome sequence generation, DNA barcodes were also used alongside the initial barcoding data for sample tracking through the genome production pipeline at the Wellcome Sanger Institute ( Twyford *et al.*, 2024). The standard operating procedures for the Darwin Tree of Life barcoding have been deposited on protocols.io ( Beasley *et al.*, 2023).

The genome size was estimated by flow cytometry using the fluorochrome propidium iodide and following the ‘one-step’ method as outlined in Pellicer *et al.* (2021). For this species, the General Purpose Buffer (GPB) supplemented with 3% PVP and 0.08% (v/v) beta-mercaptoethanol was used for isolation of nuclei ( Loureiro *et al.*, 2007), and the internal calibration standard was *Solanum lycopersicum* ‘Stupiké polní rané’ with an assumed 1C-value of 968 Mb ( Doležel *et al.*, 2007).

### Nucleic acid extraction

The workflow for high molecular weight (HMW) DNA extraction at the WSI Tree of Life Core Laboratory includes a sequence of procedures: sample preparation and homogenisation, DNA extraction, fragmentation and purification. Detailed protocols are available on protocols.io ( Denton *et al.*, 2023). In sample preparation, the ddEupPepu3 sample was weighed and dissected on dry ice ( Jay *et al.*, 2023) and leaf tissue was cryogenically disrupted using the Covaris cryoPREP ^®^ Automated Dry Pulverizer ( Narváez-Gómez *et al.*, 2023).

HMW DNA was extracted using the Automated Plant MagAttract v2 protocol ( Todorovic *et al.*, 2023). HMW DNA was sheared into an average fragment size of 12–20 kb in a Megaruptor 3 system ( Bates *et al.*, 2023). Sheared DNA was purified by solid-phase reversible immobilisation, using AMPure PB beads to eliminate shorter fragments and concentrate the DNA ( Oatley *et al.*, 2023). The concentration of the sheared and purified DNA was assessed using a Nanodrop spectrophotometer and Qubit Fluorometer and Qubit dsDNA High Sensitivity Assay kit. Fragment size distribution was evaluated by running the sample on the FemtoPulse system.

### Hi-C sample preparation

Hi-C data were generated from leaf tissue of the ddEupPepu3 sample at the WSI Scientific Operations core, using the Arima-HiC v2 kit. Tissue was finely ground using cryoPREP, and then subjected to nuclei isolation using a modified protocol of the Qiagen QProteome Kit. After isolation, the nuclei were fixed, and the DNA crosslinked using a 37% formaldehyde solution (final concentration 2%). The crosslinked DNA was then digested using the restriction enzyme master mix. The 5’-overhangs were then filled in and labelled with biotinylated nucleotides and proximally ligated. An overnight incubation was carried out for enzymes to digest remaining proteins and for crosslinks to reverse. A clean up was performed with SPRIselect beads prior to library preparation. DNA concentration was quantified using the Qubit Fluorometer v2.0 and Qubit HS Assay Kit according to the manufacturer’s instructions.

### Library preparation and sequencing

Library preparation and sequencing were performed at the WSI Scientific Operations core.

***PacBio HiFi***

At a minimum, samples were required to have an average fragment size exceeding 8 kb and a total mass over 400 ng to proceed to the low input SMRTbell Prep Kit 3.0 protocol (Pacific Biosciences, California, USA). Libraries were prepared using the SMRTbell Prep Kit 3.0 (Pacific Biosciences, California, USA) as per the manufacturer’s instructions. The kit includes the reagents required for end repair/A-tailing, adapter ligation, post-ligation SMRTbell bead cleanup, and nuclease treatment. Following the manufacturer’s instructions, size selection and clean up was carried out using diluted AMPure PB beads (Pacific Biosciences, California, USA). DNA concentration was quantified using the Qubit Fluorometer v4.0 (Thermo Fisher Scientific) with Qubit 1X dsDNA HS assay kit and the final library fragment size analysis was carried out using the Agilent Femto Pulse Automated Pulsed Field CE Instrument (Agilent Technologies) and gDNA 55kb BAC analysis kit.

Samples were sequenced on a Sequel IIe instrument (Pacific Biosciences, California, USA). The concentration of the library loaded onto the Sequel IIe was in the range 40–135 pM. The SMRT link software, a PacBio web-based end-to-end workflow manager, was used to set-up and monitor the run, as well as perform primary and secondary analysis of the data upon completion.

***Hi-C***

For Hi-C library preparation, DNA was fragmented to a size of 400 to 600 bp using a Covaris E220 sonicator. The DNA was then enriched, barcoded, and amplified using the NEBNext Ultra II DNA Library Prep Kit (New England Biolabs) following manufacturer’s instructions. Hi-C sequencing was performed using paired-end sequencing with a read length of 150 bp on an Illumina NovaSeq 6000 instrument.

### Genome assembly, curation and evaluation

***Assembly***

The HiFi reads were first assembled using Hifiasm ( Cheng *et al.*, 2021) with the --primary option. Haplotypic duplications were identified and removed using purge_dups ( Guan *et al.*, 2020). The Hi-C reads were mapped to the primary contigs using bwa-mem2 ( Vasimuddin *et al.*, 2019). The contigs were further scaffolded using the provided Hi-C data ( Rao *et al.*, 2014) in YaHS ( Zhou *et al.*, 2023) using the --break option. The scaffolded assemblies were evaluated using Gfastats ( Formenti *et al.*, 2022), BUSCO ( Manni *et al.*, 2021) and MerquryFK ( Rhie *et al.*, 2020). The organelle genomes were assembled using OATK ( Zhou *et al.*, 2024).

***Curation***

The assembly was decontaminated using the Assembly Screen for Cobionts and Contaminants (ASCC) pipeline (article in preparation). Flat files and maps used in curation were generated in TreeVal ( Pointon *et al.*, 2023). Manual curation was primarily conducted using PretextView ( Harry, 2022), with additional insights provided by JBrowse2 ( Diesh *et al.*, 2023) and HiGlass ( Kerpedjiev *et al.*, 2018). Scaffolds were visually inspected and corrected as described by Howe *et al*. (2021). Any identified contamination, missed joins, and mis-joins were corrected, and duplicate sequences were tagged and removed. The process is documented at https://gitlab.com/wtsi-grit/rapid-curation (article in preparation).

***Evaluation of the final assembly***

The Merqury.FK tool ( Rhie *et al.*, 2020), run in a Singularity container ( Kurtzer *et al.*, 2017), was used to evaluate *k*-mer completeness and assembly quality for the primary and alternate haplotypes using the *k*-mer databases ( *k* = 31) computed prior to genome assembly. The analysis outputs included assembly QV scores and completeness statistics.

A Hi-C contact map was generated for the final version of the assembly. The Hi-C reads were aligned using bwa-mem2 ( Vasimuddin *et al.*, 2019) and the alignment files were combined using SAMtools ( Danecek *et al.*, 2021). The Hi-C alignments were converted into a contact map using BEDTools ( Quinlan & Hall, 2010) and the Cooler tool suite ( Abdennur & Mirny, 2020). The contact map was visualised in HiGlass ( Kerpedjiev *et al.*, 2018).

The blobtoolkit pipeline is a Nextflow port of the previous Snakemake Blobtoolkit pipeline ( Challis *et al.*, 2020). It aligns the PacBio reads in SAMtools and minimap2 ( Li, 2018) and generates coverage tracks for regions of fixed size. In parallel, it queries the GoaT database ( Challis *et al.*, 2023) to identify all matching BUSCO lineages to run BUSCO ( Manni *et al.*, 2021). For the three domain-level BUSCO lineages, the pipeline aligns the BUSCO genes to the UniProt Reference Proteomes database ( Bateman *et al.*, 2023) with DIAMOND ( Buchfink *et al.*, 2021) blastp. The genome is also split into chunks according to the density of the BUSCO genes from the closest taxonomic lineage, and each chunk is aligned to the UniProt Reference Proteomes database with DIAMOND blastx. Genome sequences with no hits are chunked with seqtk and aligned to the NT database with blastn ( Altschul *et al.*, 1990). The blobtools suite combines all these outputs into a blobdir for visualisation.

The blobtoolkit pipeline was developed using nf-core tooling ( Ewels *et al.*, 2020) and MultiQC ( Ewels *et al.*, 2016), relying on the Conda package manager, the Bioconda initiative ( Grüning *et al.*, 2018), the Biocontainers infrastructure ( da Veiga Leprevost *et al.*, 2017), as well as the Docker ( Merkel, 2014) and Singularity ( Kurtzer *et al.*, 2017) containerisation solutions.

Table 4 contains a list of relevant software tool versions and sources.

**Table 4.** Software tools: versions and sources.

Software tool	Version	source
BEDTools	2.30.0	https://github.com/arq5x/bedtools2
BLAST	2.14.0	ftp://ftp.ncbi.nlm.nih.gov/blast/executables/blast+/
BlobToolKit	4.3.9	https://github.com/blobtoolkit/blobtoolkit
BUSCO	5.5.0	https://gitlab.com/ezlab/busco
bwa-mem2	2.2.1	https://github.com/bwa-mem2/bwa-mem2
Cooler	0.8.11	https://github.com/open2c/cooler
DIAMOND	2.1.8	https://github.com/bbuchfink/diamond
fasta_windows	0.2.4	https://github.com/tolkit/fasta_windows
FastK	427104ea91c78c3b8b8b49f1a7d6bbeaa869ba1c	https://github.com/thegenemyers/FASTK
Gfastats	1.3.6	https://github.com/vgl-hub/gfastats
GoaT CLI	0.2.5	https://github.com/genomehubs/goat-cli
Hifiasm	0.16.1-r375	https://github.com/chhylp123/hifiasm
HiGlass	44086069ee7d4d3f6f3f0012569789ec138f42b84 aa44357826c0b6753eb28de	https://github.com/higlass/higlass
Merqury.FK	d00d98157618f4e8d1a9190026b19b471055b22e	https://github.com/thegenemyers/MERQURY.FK
MultiQC	1.14, 1.17, and 1.18	https://github.com/MultiQC/MultiQC
Nextflow	23.04.0-5857	https://github.com/nextflow-io/nextflow
OATK	1	https://github.com/c-zhou/oatk
PretextView	0.2.5	https://github.com/sanger-tol/PretextView
purge_dups	1.2.3	https://github.com/dfguan/purge_dups
samtools	1.16.1, 1.17, and 1.18	https://github.com/samtools/samtools
sanger-tol/ ascc	-	https://github.com/sanger-tol/ascc
sanger-tol/ blobtoolkit	0.5.1	https://github.com/sanger-tol/blobtoolkit
Seqtk	1.3	https://github.com/lh3/seqtk
Singularity	3.9.0	https://github.com/sylabs/singularity
TreeVal	1.2.0	https://github.com/sanger-tol/treeval
YaHS	yahs-1.1.91eebc2	https://github.com/c-zhou/yahs

### Wellcome Sanger Institute – Legal and Governance

The materials that have contributed to this genome note have been supplied by a Darwin Tree of Life Partner. The submission of materials by a Darwin Tree of Life Partner is subject to the **‘Darwin Tree of Life Project Sampling Code of Practice’**, which can be found in full on the Darwin Tree of Life website here. By agreeing with and signing up to the Sampling Code of Practice, the Darwin Tree of Life Partner agrees they will meet the legal and ethical requirements and standards set out within this document in respect of all samples acquired for, and supplied to, the Darwin Tree of Life Project.

Further, the Wellcome Sanger Institute employs a process whereby due diligence is carried out proportionate to the nature of the materials themselves, and the circumstances under which they have been/are to be collected and provided for use. The purpose of this is to address and mitigate any potential legal and/or ethical implications of receipt and use of the materials as part of the research project, and to ensure that in doing so we align with best practice wherever possible. The overarching areas of consideration are:

•   Ethical review of provenance and sourcing of the material

•   Legality of collection, transfer and use (national and international)

Each transfer of samples is further undertaken according to a Research Collaboration Agreement or Material Transfer Agreement entered into by the Darwin Tree of Life Partner, Genome Research Limited (operating as the Wellcome Sanger Institute), and in some circumstances other Darwin Tree of Life collaborators.

## Data Availability

European Nucleotide Archive: Euphorbia peplus. Accession number PRJEB52211; https://identifiers.org/ena.embl/PRJEB52211. The genome sequence is released openly for reuse. The *Euphorbia peplus* genome sequencing initiative is part of the Darwin Tree of Life (DToL) project. All raw sequence data and the assembly have been deposited in INSDC databases. The genome will be annotated using available RNA-Seq data and presented through the Ensembl pipeline at the European Bioinformatics Institute. Raw data and assembly accession identifiers are reported in Table 1.

## Author information

Members of the Royal Botanic Gardens Kew Genome Acquisition Lab are listed here: https://doi.org/10.5281/zenodo.12625079.

Members of the Plant Genome Sizing collective are listed here: https://doi.org/10.5281/zenodo.7994306.

Members of the Darwin Tree of Life Barcoding collective are listed here: https://doi.org/10.5281/zenodo.12158331

Members of the Wellcome Sanger Institute Tree of Life Management, Samples and Laboratory team are listed here: https://doi.org/10.5281/zenodo.12162482.

Members of Wellcome Sanger Institute Scientific Operations: Sequencing Operations are listed here: https://doi.org/10.5281/zenodo.12165051.

Members of the Wellcome Sanger Institute Tree of Life Core Informatics team are listed here: https://doi.org/10.5281/zenodo.12160324.

Members of the Tree of Life Core Informatics collective are listed here: https://doi.org/10.5281/zenodo.12205391.

Members of the Darwin Tree of Life Consortium are listed here: https://doi.org/10.5281/zenodo.4783558.
