# Supplementary material for: The genome sequence of petty spurge, Euphorbia peplus L. (Euphorbiaceae)
Source: Wellcome Open Res. 2025 Apr 25;10:219. [Version 1] doi: 10.12688/wellcomeopenres.24030.1 (PMC12125573; doi:10.12688/wellcomeopenres.24030.1)
Supplement: Peer review history

# Reviewer report: Reviewer response for version 1

**DOI:** 10.21956/wellcomeopenres.26512.r122868

**Published:** 2025-06-03

**Authors:** Bimal K Chetri

**Affiliations:** 1 Indian Institute of Technology, Guwahati, Assam, India

This is a strong paper from the Darwin Tree of Life Consortium, presenting an impressive chromosome-level genome assembly for *Euphorbia peplus*. The authors clearly explain why sequencing this species is important, given its medicinal uses and its widespread weedy nature. The methods are well-described, covering everything from sample collection to the detailed assembly and evaluation steps.

 The quality metrics (like the scaffold N50 of 33.77 Mb, 99.54% mapping to 8 chromosomes, QV of 54.2, and 97.6% BUSCO completeness) are excellent and show this is a high-quality resource that meets EBP standards. Making the data and interactive figures readily available is also a big plus for the research community, as is the clear information on the organelle genomes. This work is a valuable contribution to the Darwin Tree of Life project and will be a key resource for future studies on Euphorbiaceae, latex, and important plant chemicals.

 I have a few points for clarification regarding the manual curation:

1. The paper says manual curation "improved" the assembly. However, it then states this "improvement" made the assembly have many more scaffold pieces (up by 90.91%) and a much lower scaffold N50 (down by 46.68%). This is a bit confusing because usually, an "improvement" means you get fewer, longer scaffolds (a higher N50). Could you please explain if the "improvement" here mainly means you fixed errors to make the assembly more accurate, even if that meant breaking up some of the longer, but incorrect, scaffolds?
2. You also mention correcting "missing joins." It would be helpful if you could briefly explain how fixing these "missing joins" also made the scaffold count go up. Normally, fixing a missing join would connect pieces, which should make the scaffold count go down.
3. While the final assembly quality shown in Table 2 looks very good (with great contig N50, BUSCO scores, etc.), the part about manual curation could use a bit more explanation. Specifically, helping us understand the impact on how connected the scaffolds are would make it clearer why this step was a good trade-off, even if it made the assembly look more fragmented in those specific stats.

Are sufficient details of methods and materials provided to allow replication by others?

Yes

Is the rationale for creating the dataset(s) clearly described?

Yes

Are the datasets clearly presented in a useable and accessible format?

Yes

Are the protocols appropriate and is the work technically sound?

Yes

Reviewer Expertise:

Organellar genome and molecular phylogeny

I confirm that I have read this submission and believe that I have an appropriate level of expertise to confirm that it is of an acceptable scientific standard.

# Reviewer report: Reviewer response for version 1

**DOI:** 10.21956/wellcomeopenres.26512.r123308

**Published:** 2025-05-30

**Authors:** Quentin Cronk, Kaede Hirabayashi

**Affiliations:** 1 University of British Columbia, Vancouver, Canada

(1) This is an informative and well-written paper. We like the interactive Hi-C map, which was useful in reviewing the assembly quality (HiGlass). The authors present a contiguous assembly with very few gaps, which is a good achievement. We appreciate the detailed explanation in the Methods section of what Blobtools is doing, as it is difficult to determine just by looking at Figures 2, 3, and 4.

 (2) We have not checked the voucher herbarium specimen, but the identification is obviously correct, based on the photographic voucher (Figure 1).

 (3) There is some discrepancy between the 277 Mb genome size reported, and the Genomescope estimate from the PacBio reads of 355 Mb. This might be worth commenting on (some discrepancy is to be expected, but this is rather large).

 (4) The presence of a repetitive-looking sequence at the gap inside chromosome/scaffold 4 (if you zoom into the Hi-C map) is concerning. It might be best to consider removing the repetitive region from the scaffold, as it is located at the gap. At a minimum, we suggest including a comment in the text. Compared to the repetitive section in scaffold 8, which has a clear interaction with only that one scaffold, this repeat appears not to indicate a very strong interaction with scaffold 4 specifically, but rather it’s present throughout the genome.

 (5) Purge_dups was used for purging duplicates, etc., in this paper. Please state whether any manual checks were involved to set the thresholds or whether only automatic thresholds were used. It isn’t mentioned in the Methods. In our experience, this tool doesn’t perform well when run with the automatic thresholds. Setting the values manually helps prevent over-purging sequences.

Are sufficient details of methods and materials provided to allow replication by others?

Yes

Is the rationale for creating the dataset(s) clearly described?

Yes

Are the datasets clearly presented in a useable and accessible format?

Yes

Are the protocols appropriate and is the work technically sound?

Yes

Reviewer Expertise:

botany and genomics

We confirm that we have read this submission and believe that we have an appropriate level of expertise to confirm that it is of an acceptable scientific standard.

# Reviewer report: Reviewer response for version 1

**DOI:** 10.21956/wellcomeopenres.26512.r123307

**Published:** 2025-05-28

**Authors:** Thomas Brazier

**Affiliations:** 1 University of Rennes, Rennes, France

Christenhusz et al. report on the assembly of a reference genome for *Euphorbia peplus*, the petty spurge. They provide a brief background on the life history, distribution, and medical importance of the species, as the plant produces a cytotoxic milky latex used in cancer treatment. They also describe with details the sampling, DNA extraction and library preparation, as well as the bioinformatics protocols for assembling a chromosome-level genome assembly for the species from long-read PacBio HiFi data and short-read Hi-C data.

 The study seems to follow modern and scientifically sound methods for collecting tissues, conducting lab work, and conducting de novo assembly of a reference genome, following the standards of the Darwin Tree of Life project. The summary statistics of the final assembly are above the minimum standards.

 I answered ‘partly’ to the question “Are sufficient details of methods and materials provided to allow replication by others?”. Globally, I think someone could easily replicate the work done here from their descriptions. However, at some point manual curation was done and not well described. In the 'Assembly statistics subsection', the authors say "The assembly was improved by manual curation, which corrected 5 misjoins or missing joins." The authors should give more details: why and how?

 Moreover, the Assembly Quality metrics QV, k-mer recovery and EBP reference standards should be briefly described (how they are computed? how to interpret them?) and compared with the minimum standard expected for a good quality assembly.

 In addition, I don't think "run in a Singularity container (Kurtzer et al., 2017)," and "The blobtoolkit pipeline was developed using nf-core tooling (Ewels et al., 2020) and MultiQC (Ewels et al., 2016), relying on the Conda package manager, the Bioconda initiative (Grüning et al., 2018), the Biocontainers infrastructure (da Veiga Leprevost et al., 2017), as well as the Docker (Merkel, 2014) and Singularity (Kurtzer et al., 2017) containerisation solutions." are necessary to understand and reproduce the study. It is good to cite tools, but these are the internal architecture of the pipeline used and are already included in the blobtoolkit citation.

 Overall it is a good genome note with sufficient details to reproduce the experiment and evaluate the quality of the assembly.

Are sufficient details of methods and materials provided to allow replication by others?

Partly

Is the rationale for creating the dataset(s) clearly described?

Yes

Are the datasets clearly presented in a useable and accessible format?

Yes

Are the protocols appropriate and is the work technically sound?

Yes

Reviewer Expertise:

bioinformatics, population genomics

I confirm that I have read this submission and believe that I have an appropriate level of expertise to confirm that it is of an acceptable scientific standard.
